# Physician and Patient Adjustment to Reference Pricing for Drugs

**DOI:** 10.1001/jamanetworkopen.2019.20544

**Published:** 2020-02-05

**Authors:** James C. Robinson, Christopher Whaley, Timothy T. Brown, Sanket S. Dhruva

**Affiliations:** 1 University of California School of Public Health, Berkeley; 2 RAND Corporation, Santa Monica, California; 3 Department of Medicine, University of California School of Medicine, San Francisco; 4 San Francisco Veterans Affairs Medical Center, San Francisco, California

## Abstract

**Importance:**

Reference pricing has been shown to reduce drug spending in Europe and has been adopted by some employers and labor unions in the United States. Its association with patient cost sharing depends on whether and how quickly physicians adjust their prescribing patterns to favor the least costly alternatives within each therapeutic class.

**Objective:**

To examine whether the implementation of reference pricing is associated with physicians and patients shifting to lower-cost drugs, thereby reducing consumer cost sharing and the prices paid by employers.

**Design, Setting, and Participants:**

This economic evaluation included employees of Catholic organizations who purchased health insurance through the Reta Trust and a random sample of employees of public sector organizations who purchased insurance through the California Public Employees’ Retirement System (CalPERS) as a comparison group between July 1, 2010, and December 31, 2017. Data analysis was performed from January 1, 2019, to September 1, 2019.

**Exposures:**

The Reta Trust implemented reference pricing in July 2013; CalPERS did not adopt reference pricing during the study period.

**Main Outcomes and Measures:**

Probability that the drug prescribed was the least costly alternative within its therapeutic class, price paid per prescription, and patient cost sharing per prescription. Multivariable, difference-in-differences regression analysis of drug insurance claims was performed for patients before and after implementation of reference pricing, adjusted for patient characteristics, each drug’s therapeutic class, and the month and year of the prescription.

**Results:**

During the study period, a total of 1.2 million prescriptions were submitted by 34 319 individuals covered by Reta Trust and 2.1 million prescriptions were submitted by 738 159 individuals covered by CalPERS. In the first 2.5 years after implementation of reference pricing, the percentage of prescriptions made for the low-priced drug within each therapeutic class increased by 5.1 percentage points (95% CI, 1.8 to 8.4 percentage points), patient cost sharing increased by 10.3% (95% CI, −1.6% to −23.6%; this difference was not statistically significant), and prices paid decreased by 19.1% (95% CI, −30.2% to −6.2%) for Reta Trust patients compared with CalPERS patients. During the subsequent 2-year postimplementation period, the percentage of prescriptions made for the low-priced drug increased an additional 6.2 percentage points (95% CI, 2.3 to 10.1 percentage points), patient cost sharing decreased by 21.3% (95% CI, −31.2% to −9.9%), and prices paid increased by 7.2% (95% CI, −12.6% to 31.4%; this difference was not statistically significant). Relative to the change experienced by the CalPERS population, during the study period, the share of prescriptions for lower-priced drugs increased by 6.3 percentage points (8.9% relative increase), the mean prescription drug price decreased by $9.5 (12.1% relative decrease), and the mean patient cost sharing decreased by $1.8 (4.3% relative decrease).

**Conclusions and Relevance:**

In this study, reference pricing was associated with a combination of lower prices paid by employers and lower cost sharing by employees but with a time lag in prescribing habits by physicians.

## Key Points

**Question:**

Is reference pricing in employment-based health insurance associated with prescribing lower-priced drugs, and is this prescribing practice associated with reductions in cost sharing by patients?

**Findings:**

In this economic evaluation of 3.3 million drug insurance claims, after implementation of reference pricing, physicians increased the prescription of the low-cost drugs within each therapeutic class, and this increase was associated with a reduction of prices paid by employers and cost sharing paid by employees.

**Meaning:**

Reference pricing may shift the mix of drugs dispensed from those offering the highest rebates to the pharmacy benefit manager to those offering the lowest prices to the employer and employee.

## Teaser

This economic evaluation of drug insurance claims examines whether the implementation of reference pricing is associated with physicians and patients shifting to lower-cost drugs, thereby reducing consumer cost sharing and the prices paid by employers.

## Introduction

Reference pricing is a strategy developed by European nations and adopted by some US employers that encourages physicians to prescribe and patients to use the least costly medications within therapeutic classes that feature multiple alternatives.^1,2^ Under reference pricing, the employer’s or insurer’s payment is limited to the price of the least costly product in each therapeutic class. Patients using higher-priced drugs within the class must pay the full difference themselves, unless the prescribing physician requests an exemption on clinical grounds. This approach creates incentives for physicians to switch their prescriptions to lower-priced products on behalf of their patients, in turn generating savings for the employer or insurer.

Reference pricing has been applied only recently and in limited contexts in the United States. A study^3^ reported an increase in the percentage of prescriptions filled for the lowest-cost alternatives and an associated reduction in the mean prices paid by employers. However, the savings to employers were accompanied by increased cost sharing for patients, who faced higher out-of-pocket spending on those prescriptions still written by their physicians for high-priced drugs. The published results were based on trends in drug prescribing and dispensing in the first 18 months after implementing reference pricing compared with the 24 months before implementation with trends in a similar employed population that did not implement reference pricing during the period under analysis. In the current study, we extended the postimplementation period from 18 to 54 months to examine whether the pattern of physician prescription and patient use adjusted over time and whether this pattern was associated with reduced patient cost sharing without loss of savings for employers.

## Methods

This economic evaluation included employees of Catholic organizations who purchased health insurance through the Reta Trust and a random sample of employees of public sector organizations who purchased insurance through the California Public Employees’ Retirement System (CalPERS) as a comparison group between July 1, 2010, and December 31, 2017. Data analysis was performed from January 1, 2019, to September 1, 2019. Patient consent was not solicited or required because the claims data were deidentified. This study was approved by the institutional review board at the University of California, Berkeley. This study followed the Consolidated Health Economic Evaluation Reporting Standards (CHEERS) reporting guideline.

### Reference Pricing

Reference pricing incorporates several structural features that differ from the more common manner by which employers and insurers manage pharmaceutical benefits. Under the common approach, pharmacy benefit managers (PBMs) assign individual drugs to one of several tiers within their contracted formularies, usually based on the drug’s price. Generic drugs typically are assigned to the first tier, branded drugs that offer substantial rebates are assigned to the second tier, and branded drugs that offer little to no rebates are assigned to the third tier. Consumer co-payments are lowest in tier 1 (eg, $10), moderate in tier 2 (eg, $30), and high in tier 3 (eg, $75). The PBMs authorize pharmacies to automatically substitute less expensive drugs for more expensive drugs (especially generics for brands) without consulting the prescribing physician. In contrast, under reference pricing, drugs are grouped by therapeutic class, and the lowest-priced drugs are available to the patient for a low co-payment (eg, $10 in the case of the Reta Trust). For other drugs, patients must pay this standard co-payment plus the difference between the price of the chosen drug and that of the low-priced alternative. Pharmacists do not switch one drug to another without consulting with the prescribing physician. Changes in the selection of drugs dispensed and reimbursed through insurance claims after implementation of reference pricing therefore reflect changes in physician prescription patterns and not a substitution at the pharmacy without physician intervention.

The pattern of drugs selected and prices paid reflect the contracts negotiated by the PBMs with pharmaceutical manufacturers and distributors. Different PBMs have somewhat different sets of drugs covered in their formularies. During the study period, the Reta Trust switched PBMs, exposing employees to a new pattern of cost sharing. For example, a patient who was using a low-priced drug and thus paying only the standard $10 co-payment could be faced with a higher co-payment after the change in PBMs if that drug were not the low-priced alternative for the new PBM. The patient and prescribing physician would need to switch to the new low-priced alternative to avoid the higher cost sharing. Changes in PBMs are decided by employers and insurers based on nontransparent rebates and other financial considerations but can create substantial confusion and financial obligations for employees.

### Claims Data

The claims data used in this study extend from July 1, 2010, through December 30, 2017. The Reta Trust implemented reference pricing in July 2013, whereas CalPERS did not implement reference pricing and served as the comparison group. In January 2016, the Reta Trust switched contracting PBMs. This switch would be expected to have an adverse short-term effect on Reta Trust employers because they would face higher prices for drugs that were the low-cost alternatives in the previous PBM’s formulary but were higher priced in the new PBM’s formulary. Different PBMs obtain different prices for similar drugs based on their contracting practices. CalPERS did not change contracting PBMs during the study period.

The claims data cover 2070 distinct drugs, both branded and generics, that fall into 70 therapeutic classes. The classes are defined by the American Hospital Formulary Service Pharmacologic-Therapeutic Classification System, which also is used to classify drugs for Medicaid and Medicare Part D formularies. The purchasing alliance did not include expensive specialty drugs managed through the pharmacy benefit or infused biologics managed through the medical benefit in its reference pricing program. We did not have access to pharmacy use and spending data for CalPERS employees enrolled in the Kaiser Permanente health plan, which manages its own internal pharmacy program.

### Statistical Analysis

We calculated trends in the percentage of prescriptions made for the lowest-price (reference) drug within its therapeutic class, the mean price paid per prescription by the employer alliance (Reta Trust or CalPERS), and the mean cost sharing paid by the patient. We compared levels and trends in the study end points before and after the implementation of reference pricing by the Reta Trust.

We then conducted multivariable difference-in-differences regression analyses to measure the association between implementation of reference pricing at RETA and each of the study end points, adjusting for marketwide trends in pharmaceutical prices that are captured in the CalPERS data and changes in the demographic characteristics of the RETA and CalPERS populations. Difference-in-differences methods are used with observational data to approximate the study design of controlled trials, comparing trends for individuals in the treatment and comparison groups.^4^ We divided the postimplementation period into the first 2.5 years (July 1, 2013, through December 31, 2015) and the subsequent 2 years (January 1, 2016, through December 31, 2017) to distinguish short-term from longer-term physician and patient responses. Statistical analyses were performed using Stata, version 15 (StataCorp).

A key assumption of the difference-in-differences analyses is that the trends of each dependent variable were parallel across the treatment and comparison groups before the implementation of reference pricing. We tested the validity of this assumption by examining whether these trends were statistically parallel in each quarter before the implementation of reference pricing. Statistical tests of the trends of each dependent variable support the assumption of parallel trends before the implementation of reference pricing.

## Results

During the study period, 34 319 individuals received pharmaceutical benefits through the Reta Trust and 738 159 through CalPERS. We obtained 1.2 million drug claims from the Reta Trust, a health care purchasing alliance of Catholic organizations, and a random sample of 2.1 million claims from CalPERS, a purchasing alliance of public agencies. Both purchasing alliances are self-insured and thus maintain their own claims while working with contracting PBMs to administer their pharmacy benefits. Reference pricing was applied only to patient self-administered drug classes managed within the pharmacy benefit and not to infused and injected biologics managed through the medical benefit.

Table 1 presents descriptive statistics for the Reta Trust and CalPERS populations for the preimplementation period, first 2.5 years after implementation, and the second postimplementation period. During the preimplementation period, the Reta Trust population had a lower share of prescriptions for low-priced drugs than the CalPERS population (69.5% vs 77.1%). This difference narrowed after implementation (74.0% vs 76.5% during the second postimplementation period). Similar trends were observed for mean prescription paid prices and mean patient cost-sharing payments per prescription.

**Table 1.** Descriptive Statistics of the Study Participants

Variable	July 2010 to June 3 2013		First Postimplementation Period (July 1, 2013, to December 31, 2015)		Second Postimplementation Period (January 1, 2016, to December 31, 2017)	
	Reta Trust	CalPERS	Reta Trust	CalPERS	Reta Trust	CalPERS
No. of covered individuals	20 434	369 939	16 249	431 461	16 153	386 497
No. of prescriptions	510 158	631 516	349 930	793 435	339 475	664 042
Prescriptions for low-priced drugs, mean (SD), %	69.5 (46.0)	77.1 (42.0)	74.0 (43.9)	76.5 (42.4)	74.3 (43.7)	75.5 (43.0)
Price paid per prescription, mean (SD), $	85.7 (149.6)	74.0 (170.2)	76.8 (194.2)	88.0 (270.6)	81.8 (227.4)	79.5 (245.6)
Consumer cost sharing per prescription, mean (SD), $	17.6 (20.1)	12.2 (16.0)	18.5 (36.8)	11.2 (19.8)	13.4 (33.3)	9.8 (23.5)

Abbreviation: CalPERS, California Public Employees Retirement System.

The Figure presents trends in the cost sharing per prescription at the Reta Trust and CalPERS. In the period before reference pricing, mean (SD) patient cost sharing payments were $17.6 ($20.1) for the Reta Trust population and $12.2 ($16.0) for the CalPERS population. By the end of the study period, patient payments decreased to $13.4 ($33.3) for the Reta Trust population and increased to $9.8 ($23.5) for the CalPERS population. Cost sharing per prescription initially was substantially higher for the Reta Trust than for the CalPERS employees, reflecting the generosity of the public employee health insurance benefit design. Cost sharing by the Reta Trust employees increased in the immediate postimplementation period, further increasing the difference with the CalPERS employees. This increase was associated with some Reta Trust employees having received drugs with prices above those of the lowest-cost alternative in their therapeutic class and now being required to pay the price difference in addition to the standard $10 co-payment per prescription. However, cost sharing then decreased steadily as physicians adjusted their prescribing patterns to reduce their patients’ financial obligations. By the end of the study period, cost sharing at the Reta Trust was still decreasing and was below the levels experienced before reference pricing, although still not as low as cost sharing by the CalPERS employees.

**Figure.**
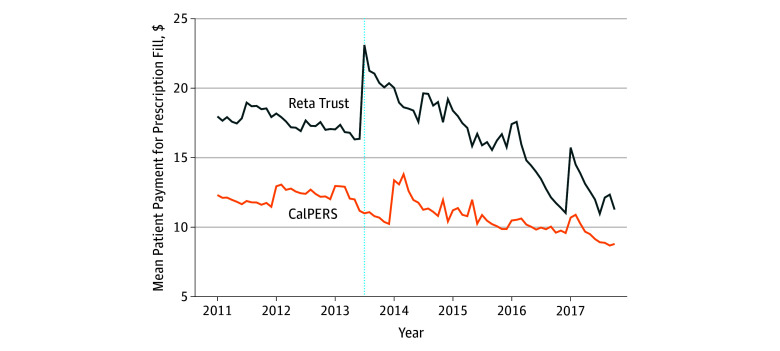
Patient Cost Sharing Trends Dashed line indicates the time of implementation of reference pricing (July 1, 2013). CalPERS indicates California Public Employees’ Retirement System.

Table 2 presents results from the multivariable, difference-in-differences statistical analyses. In the preimplementation period, Reta Trust employees used 5.8 percentage points fewer low-priced drugs (95% CI, −9.4 to −2.2 percentage points) and paid 33.8% higher cost sharing per prescription (95% CI, 23.9% to 44.5%), whereas Reta Trust employers paid prices 36.7% higher than those paid by CalPERS employers (95% CI, 14.2% to 63.5%).

**Table 2.** Multivariable Difference-in-Differences Regression Results Comparing Trends in Drug Selection, Patient Cost Sharing, and Mean Prices Paid for the Reta Trust and the California Public Employees Retirement System

Variable	Use of Low-Priced Drugs, Mean (95% CI), Percentage Points	Patient Cost Sharing, Mean (95% CI), %	Drug Price, Mean (95% CI), %
Difference between Reta Trust and CalPERS			
Before implementation of reference pricing	−0.0579 (−0.0936 to −0.0222)	33.79 (23.86 to 44.51)	36.67 (14.24 to 63.50)
First 30 mo after implementation^a^	0.0510 (0.0182 to 0.0837)	10.26 (−1.592 to 23.55)	−19.07 (−30.18 to −6.182)
Months 31-54 after implementation^b^	0.0621 (0.0229 to 0.101)	−21.28 (−31.23 to −9.882)	7.156 (−12.60 to 31.37)
Drug price market trends			
First 30 mo after implementation^a^	−0.0148 (−0.0490 to 0.0194)	−19.79 (−28.85 to −9.577)	−10.29 (−18.63 to −1.083)^c^
Months 31-52 after implementation^b^	−0.0215 (−0.0680 to 0.0250)	−30.57 (−38.50 to −21.63)	−35.05 (−45.53 to −22.56)^a^
Observations, No.	3 288 852	3 288 852	3 288 852
*R* ^2^	0.302	0.258	0.379

^a^ From July 1, 2013, to December 31, 2015.

^b^ From January 1, 2016, to December 31, 2017.

This pattern changed after implementation of reference pricing. In the first 2.5 years after implementation, the percentage of prescriptions made for the low-priced drug within each therapeutic class increased by 5.1 percentage points (95% CI, 1.8 to 8.4 percentage points), patient cost sharing increased by 10.3% (95% CI, −1.6% to −23.6%; this difference was not statistically significant), and prices paid decreased by 19.1% (95% CI, −30.2% to −6.2%) for Reta Trust patients compared with CalPERS patients. During the subsequent 2-year postimplementation period, the percentage of prescriptions made for the low-priced drug increased an additional 6.2 percentage points (95% CI, 2.3 to 10.1 percentage points), patient cost sharing decreased by 21.3% (95% CI, −31.2% to −9.9%), and prices paid increased by a 7.2% (95% CI, −12.6% to 31.4%; this difference was not statistically significant). By the end of the study period, the percentage of prescriptions made for the low-priced drug was higher, whereas patient cost sharing and mean prices paid were lower than before implementation of reference pricing.

## Discussion

A previously published study^3^ reported that the implementation of reference pricing was followed by rapid shifts in prescriptions to the lowest-cost alternative drug in each therapeutic class, leading to substantial savings to the employers. However, those changes were accompanied by an increase in consumer cost sharing because some physicians continued to prescribe and some patients continued to use more expensive products. The current study was designed to investigate the persistence of that association over time because 3 years were added to the postimplementation follow-up period.

The implementation of reference pricing was associated with a shift in the share of prescriptions being made for the low-cost drug within therapeutic classes, and this shift continued during the longer postimplementation period. The consequent reduction in mean prices continued but was interrupted by the decision of the Reta Trust to shift to a different PBM midway through the period. This new PBM had a different set of contracts with pharmaceutical manufacturers and therefore a different set of negotiated prices. Drug prices decreased after the switch to the new PBM.

The focus of the study was on the evolution of patient cost sharing after implementation of reference pricing. We report an initial upward spike in cost sharing after implementation presumably because of a lag in response by physicians and patients to the new incentives. This finding is consistent with the higher cost sharing reported in another article.^3^ However, cost sharing then decreased steadily as physicians and patients adjusted. The longer postimplementation period in this new analysis allowed us to observe the extended decrease in cost sharing from that initial spike. By the end of the study period, consumer cost sharing at the Reta Trust had decreased to a level below that paid before implementation of reference pricing (Figure and Table 2). This finding implies that the reduced drug spending by the employers from reference pricing was not associated with increased cost sharing by the employees.

These findings indicate that patients benefitted over time from the implementation of reference pricing, but the initial increase in cost sharing could have negative clinical implications. In a 2019 survey study,^5^ 29% of patients reported not taking medications as prescribed within the past year because of cost. Nonadherence is a significant national problem, and the temporary increases in cost sharing found in this study could aggravate the problem. Fortunately, the reduced out-of-pocket costs in the long term would be expected to encourage improved adherence and therefore be associated with improved clinical outcomes.

That physicians adapt quickly to the implementation of reference pricing and reduce the lag in shifting prescribing toward lower-cost therapeutic alternatives is imperative. Although high prescription drug prices and lack of affordability are firmly established in national policy conversations, physicians often miss opportunities to discuss out-of-pocket costs with patients and help patients navigate these concerns.

A solution may be afforded by the ubiquity of electronic health records. Real-time benefits tools can provide physicians with information about expected patient-specific out-of-pocket spending, relying on information derived from pharmacy benefit plans.^6^ The Centers for Medicare & Medicaid Services finalized a rule requiring Medicare Part D plan sponsors to implement at least 1 real-time benefits tool that can integrate with an electronic health record or electronic prescribing system of its choosing by the start of 2021, with encouragement to do so sooner. Such tools could provide point-of-care clinical information that inspires physician-patient conversations and facilitates prescriptions of low-cost drug alternatives.

An additional strategy would be to provide prescribers with feedback on patients’ costly medications when cheaper alternatives are available. Because physicians often care for patients in multiple different health plans, such data would help increase awareness and likely lead to prescribing of lower-cost alternatives and applications for exemptions on clinical grounds if those alternatives were not thought to be appropriate; both actions would reduce the time during which some patients encounter higher cost sharing. In the long term, increasing use of reference pricing across more health plans would be expected to lead physicians to adapt more quickly because of their experience and propensity to prescribe lower-cost therapeutic alternatives. Both health plans and patients would benefit from cost savings, with associated higher adherence and improved clinical outcomes for patients.

As a component of health insurance benefit design, reference pricing encourages but does not require the physician to change the prescription to a lower-cost alternative if available within the therapeutic class. The drug is dispensed as originally prescribed if the physician does not approve the change. Reference pricing differs from the automatic substitution of generic for branded drugs, a strategy prevalent in the United States, in that it relies on the physician to change the prescription rather than having the change made by the pharmacist without physician consultation. Automatic substitution at the pharmacy favors drugs that offer the largest rebates to the PBM, which may not be those charging the lowest prices.^7,8,9^

Under traditional cost sharing, the patient’s coinsurance and deductible responsibility is linked to the list price of the drug, not the actual price paid by the PBM after accounting for negotiated rebates. The full value of the rebate is kept by the PBM, the employer, or the insurer, without benefitting the patient. With the cooperation of the physician, reference pricing may shift the mix of drugs dispensed from those offering the highest rebates to those offering the lowest prices. Reference pricing therefore encourages drug manufacturers to negotiate low prices rather than negotiate high prices accompanied by high rebates.

The association of reference pricing with patient cost sharing will depend on the speed with which physicians respond to the new financial incentives.^10^ In this study of patients covered by employment-based insurance, cost sharing increased initially after implementation of reference pricing and then decreased, resulting in a combination of lower prices paid by the employer and lower cost sharing by the employees. The lag in physician and consumer responses to reference pricing and the transient increase in cost sharing after implementation highlight the importance of prompt and effective communication strategies so that prescribers and patients understand and can respond to the changing incentives.

### Limitations

This study has limitations. The study sample consisted of patients covered by an alliance of nonprofit organizations (Catholic dioceses that employ school, charitable, and religious workers) compared with patients covered by an alliance of public sector agencies. The study is not representative of the entire population of patients covered by employment-based insurance, such as those employed in small private firms and large for-profit corporations. The reference pricing program covered 70 classes of mostly oral, self-administered drugs obtained by patients in retail and mail-order pharmacies and did not include high-cost specialty drugs and biologics.

The study was not structured as a randomized clinical trial; however, employees of the Reta Trust were not given the opportunity to be uncovered by reference pricing once the alliance had chosen that strategy. Employees covered by CalPERS were not given the opportunity to opt into a reference pricing benefit design. We used multivariable, difference-in-differences statistical methods to compare the changes in drug selection, prices paid, and consumer cost sharing before and after implementation of reference pricing at the Reta Trust compared with the changes, if any, at CalPERS during the same period. Difference-in-differences methods ensure that changes in the larger environment not attributable to the intervention under study (implementation of reference pricing) will not be ascribed statistically to that intervention because they also affect the comparison group. The Reta Trust and CalPERS populations were similar and satisfied the parallel trends assumption of the difference-in-differences methods (eg, that, absent the intervention, the 2 groups would have performed the same throughout the entire period).

## Conclusions

In this study, reference pricing was associated with a combination of lower prices paid by employers and lower cost sharing by employees. However, there was a time lag in prescribing habits by physicians.
